# Cardio-metabolic profile is a determinant of carotid artery disease quantified by Magnetic Resonance Imaging

**DOI:** 10.1186/1532-429X-11-S1-P166

**Published:** 2009-01-28

**Authors:** Saurabh S Dhawan, Asad Ghafoor, Hamid S Syed, Christina Niessner, Konstantinos Aznaouridis, Muhammad Ali, Ibhar Al Mheid, Irina Uphoff, Charles B Kitchen, John N Oshinski, Dean P Jones, Arshed A Quyyumi

**Affiliations:** Emory University School Of Medicine, Atlanta, GA USA

**Keywords:** Body Mass Index, Metabolic Syndrome, Carotid Artery, Diastolic Blood Pressure, Common Carotid Artery

## Introduction

Magnetic Resonance Imaging (MRI) can be used to measure common carotid artery wall volume (CCA-CWV) and maximum wall thickness (CCA-CWT) that incorporates adventitial thickness in addition to intima-media thickness (CCA-IMT). Although, the predictors of CCA-IMT are well known, those of CCA-CWV and CCA-CWT have not been studied.

## Purpose

We hypothesized that patients with higher CCA-CWV and CCA-CWT have lower high-density lipoprotein (HDL) levels and greater incidence of metabolic syndrome.

## Methods

One hundred and three subjects with IMT >0.65 mm underwent MRI of their carotid arteries using T2-weighted black-blood sequence transaxial slices to measure CCA-CWV and CCA-CWT. Blood pressure (BP), body mass index (BMI), fasting lipid profile and glucose were measured. Metabolic syndrome (MetS) was defined in patients as having ≥ 3 risk factors, BMI ≥ 30, TG ≥ 150 mg/dl, HDL ≤ 40 mg/dl in men and ≤50 mg/dl in women, BP ≥ 130/85 and fasting glucose ≥ 110 mg/dl.

## Results

CCA-CWV correlated negatively with serum HDL levels (r = -0.373, p < 0.0001), positively with BMI (r = 0.54, p < 0.0001) and diastolic BP (r = 0.28, p = 0.009) independent of age, sex, the presence of diabetes, hypertension, hyperlipidemia and smoking. CCA-CWT correlated negatively with serum HDL levels (and r = -0.255, p = 0.01) and positively with presence of MetS (r = 0.23, p = 0.02) independent of age, gender and history of smoking.

## Conclusion

Cardio-metabolic profile is a predictor of carotid artery disease as quantified by magnetic resonance imaging.

